# Fluid responsiveness and venous congestion: unraveling the nuances of fluid status

**DOI:** 10.1186/s13054-024-04930-2

**Published:** 2024-04-26

**Authors:** Adrien Joseph, Matthieu Petit, Philippe Vignon, Antoine Vieillard-Baron

**Affiliations:** 1 Medical and Surgical ICU, University Hospital Ambroise Pare, GHU Paris-Saclay, AP-HP, Boulogne-Billancourt, France; 2 Inserm U1173, Laboratory of Infection and Inflammation, University Versailles Saint Quentin - University Paris Saclay, Guyancourt, France; 3 Inserm U1018, CESP, University Versailles Saint Quentin - University Paris Saclay, Guyancourt, France; 4 Medical-Surgical Intensive Care Unit, INSERM CIC 1435 and Faculty of Medicine, University of Limoges, Limoges, France

The concept of fluid tolerance, which refers to the capacity of patients to undergo fluid expansion without experiencing harmful effects of volume overload with organ dysfunction, is gaining recognition in both intensive care literature [1] and clinical practice, driven by the mounting evidence of the detrimental impacts of fluid overload [2]. It physiologically makes sense that systemic venous return can still increase even when some degree of venous congestion is already present. In the past, Patterson and Starling reported a transition phase before fluid-induced right ventricular (RV) failure, where increasing RV inflow increased RV outflow despite slightly elevated right atrial pressure (RAP) and a RV functioning above its normal unstressed volume [3].

Recently, Muñoz et al. [4] prospectively reported in 90 mechanically ventilated patients under norepinephrine infusion that markers of venous congestion frequently coexist with markers of fluid responsiveness (FR) in a similar frequency than in patients with no markers of FR. This result was not completely unexpected. In the beginning of the era of FR strategy, Osman et al. reported that among fluid-responsive patients, central venous pressure (CVP) as well as pulmonary artery occlusion pressure (PAOP) could be significantly elevated, suggesting some degree of congestion [5]. This underscores the need for intensivists to better understand the need and potential indications for fluid expansion. The concept of fluid tolerance raises pivotal questions about the clinical benefits and effect on cardiac output of fluid therapy, as well as its potential risks of worsening venous congestion even in fluid-responsive patients. In other words, it emphasizes that in shock patients, even those who are fluid-responsive, the appropriate therapeutic approach may not always involve administering more fluids.

In this comment, using original data, we highlight the ambiguity surrounding the clinical significance of congestion markers and attempt to clarify and discuss the concept of fluid tolerance. One classical marker of venous congestion is CVP, a surrogate of the RAP. Pesenti et al. [6] nicely re-emphasized how important could be its monitoring. CVP is the downstream pressure of the systemic venous return and then acts on organ perfusion and function. Chen et al. [7] reported that admission CVP was associated with increased risk of acute kidney injury in critically ill patients with an Odds ratio of 1.02 [1.00–1.03] for every cmH_2_O increase. Mullens et al. [8] reported in 145 patients with advanced decompensated heart failure that a CVP ≥ 8 mmHg after intensive medical therapy despite a normal cardiac index was much more at risk of worsening renal function than a CVP < 8 mmHg but a low cardiac index. Defining an optimal CVP threshold for venous congestion is challenging, and choosing a threshold of 12 mmHg as done by Muñoz et al. [4] may underestimate venous congestion in fluid-responsive patients. Besides the paper by Mullens et al., Boyd et al. [9] referenced in a reanalysis of the VASST trial that patients with septic shock whose CVP was 8–12 mmHg had already a higher mortality than those with CVP < 8 mmHg. In our Hemopred database with 540 invasively mechanically ventilated patients with circulatory failure [10], we explored relationship between FR, defined using the response to passive leg raising (PLR), and CVP. When applying a threshold of 12 mmHg, only 25% of fluid-responsive patients could be suspected to exhibit venous congestion, a similar rate than observed in fluid-unresponsive patients (Fig. 1, panel A). However, with a threshold of 8 mmHg, this was now more frequent in fluid-unresponsive than in fluid-responsive patients (64% vs. 52%, respectively, *p* = 0.008, Fig. 1, panel B). What is interesting to note is that frontline physicians in this study probably took into account the concept of fluid tolerance. Indeed, fluid-responsive patients with a CVP > 8 mmHg tended to receive less frequently fluid expansion than those with a CVP < 8 mmHg (57/85, 67% vs. 80/100, 80%, respectively, *p* = 0.067).

**Fig. 1:**
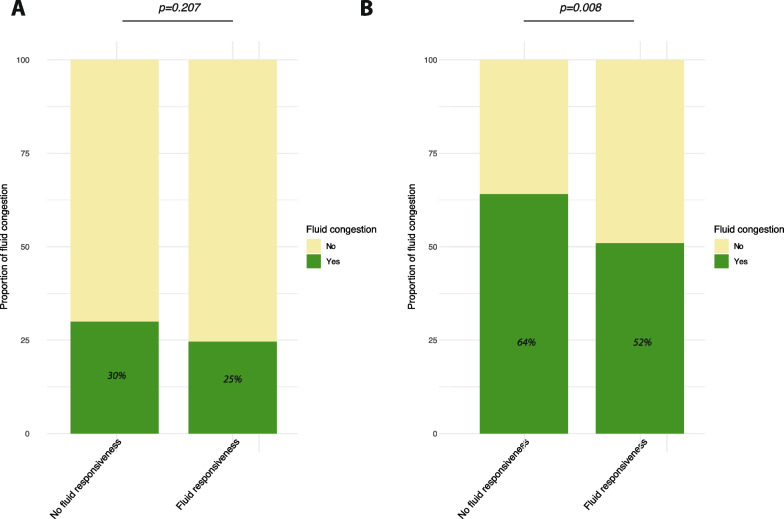
Proportion of fluid congestion in fluid responsive and non-fluid responsive patients using CVP > 12 mmHg (**A**) or CVP > 8 mmHg criterion (**B**)

Besides its threshold, another potential limitation of CVP to evaluate venous congestion is that it does not give direct information on organ perfusion. Other approaches have been proposed as the Venous EXcess Ultrasound (VExUS) score [11]. Ultrilla-Alvarez et al. [12] reported in 60 post-cardiac surgery patients that VExUS score was positively correlated with the mean systemic filling pressure. However, in a prospective observational study performed in 145 critically-ill patients, Andrei et al. [13] reported this score is far from perfect as it was not associated with acute kidney injury neither at admission nor at day 2 or day 3. In their study, Muñoz et al. defined venous congestion if at least one of the following parameters was present, a CVP > 12 mmHg, a VExUS score > 1, an E/E’ > 10 and a lung ultrasound score > 10, the 2 latter being markers of pulmonary congestion (ref). They reported 53% of patients with markers of FR exhibited markers of venous congestion, while interestingly this dropped to 25% when a CVP > 12 mmHg was only used or a VExUS score > 1.

The concept of fluid tolerance is of great interest and brings us from the simplistic view of fluid congestion and fluid responsiveness as a continuum (Fig. 2A) towards a more nuanced understanding where signals of congestion and fluid responsiveness coexist (Fig. 2B). However, our findings underline the difficulties of defining venous congestion, as opposed to fluid responsiveness, and question the significance of venous congestion signals according to predefined thresholds as well as the indication for fluid expansion in patients still in shock but with already some degree of venous congestion. One could consider that patients with direct or indirect signs of venous congestion should not be undergo fluid expansion anymore even in the presence of persistent circulatory failure, but the translation of venous congestion signals into harmful effects of fluid expansion remains an unanswered question. Future studies should delve deeper into understanding the clinical impact of these intertwined concepts, aiming to guide therapeutic strategies that optimize fluid management in critically ill patients.

**Fig. 2:**
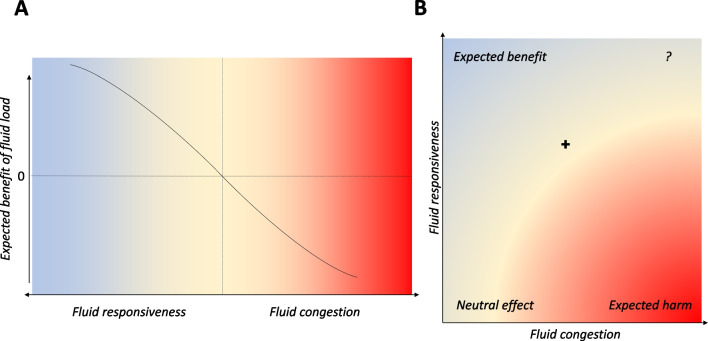
Theoretical representation of fluid responsiveness and fluid congestion. These should not be regarded as a continuum (**A**) but rather as a two-dimensional state where signals of fluid responsiveness and congestion coexist (**B**). The benefit/harm of fluid expansion does not probably follow a linear relation with volume status (**A**) but rather positions itself in a two-dimensional state (**B**)

We look forward to contributing to the ongoing dialogue in this important area of critical care.

## Data Availability

Data on which this comment is based have been published previously [10] and are available upon reasonable request.
